# Severe late‐onset neutropenia induced by ocrelizumab in a multiple sclerosis patient: A case report

**DOI:** 10.1002/ccr3.5299

**Published:** 2022-01-19

**Authors:** Robin Rauniyar, Rahul Rauniyar, Ankita Agrawal, Shikha Yadav, Sreekant Avula

**Affiliations:** ^1^ 113015 Maharajgunj Medical Campus Tribhuvan University Institute of Medicine Maharajgunj Nepal; ^2^ Internal Medicine The Wright Center for Graduate Medical Education Scranton Pennsylvania USA; ^3^ Nepal Army Institute of Health Sciences Tribhuvan University Kathmandu Nepal; ^4^ Nepalgunj Medical College Kathmandu University Kathmandu Nepal

**Keywords:** late‐onset neutropenia, multiple sclerosis, ocrelizumab

## Abstract

Ocrelizumab is a recombinant humanized antibody targeted against CD‐20 molecule, which was approved for the treatment of relapsing and primary progressive multiple sclerosis. Common adverse events of ocrelizumab include infusion‐related reactions like rash, pruritus, and flushing. Late‐onset neutropenia (LON) is a rarely reported complication of ocrelizumab therapy. We report a case of severe late‐onset neutropenia in a patient with primary progressive multiple sclerosis treated with ocrelizumab with neutropenia occurring 3 months after the last dose received treated with empirical broad‐spectrum intravenous antibiotics and filgrastim. Severe late‐onset neutropenia is a rare unpredictable adverse event and outlines the importance of regular routine blood workup for detecting severe neutropenia early in its course.

## Abstract 2

We would like to acknowledge Rohit Rauniyar for his valuable inputs during the writing of this article.

## 1 BACKGROUND

The role of ocrelizumab as a disease‐modifying therapy in the treatment of multiple sclerosis (MS) has been identified in recent times. It is a recombinant humanized antibody targeted against the CD‐20 molecule, a glycosylated phosphoprotein expressed on the surface of a range of B‐cell lineages. Thus, antibody‐mediated CD‐20 B‐cell depletion plays a major role in its action against MS.^1^ Ocrelizumab was approved by the US Food and Drug Administration (FDA) in March 2017 for relapsing forms of MS and primary progressive multiple sclerosis (PPMS).^2^ The most common side effects of the drugs in the clinical trials were infusion‐related reactions in about 34.2% of patients, which included rash, pruritus, throat irritation, and flushing,^3^ with other side effects noted to be infections and malignancies that were not surely related to the drug.^4^

In this case report, we present a patient who presented with late‐onset neutropenia (LON), a rarely reported side effect of ocrelizumab in the treatment of MS. We aim to highlight this uncommon adverse effect and emphasize the importance of regular hematologic surveillance and counseling such patients regarding the dangers of severe neutropenia.

## 2 CASE PRESENTATION

A 38‐year‐old man with a past medical history of PPMS diagnosed 6 years back presented to the emergency department with complaints of continuous, low‐grade fever, chills, rigor, and painful swelling of the left great toe. He also complained of generalized weakness and vesicular lesions in the mouth. The patient started ocrelizumab therapy 3.5 years prior with use every 6 months. He reports his last drug administration was approximately 3 months prior to presentation. He reported no history of trauma to the great toe but did note a history of MS flare‐up 2 months back. Recent significant medical events included an episode of presumed viral infection 2 months prior resulting in progressive and temporary loss of all motor functions for 1 week.

He was conscious and well oriented with ill‐looking demeanor and stable vitals. He had a grossly erythematous and tender swelling on his left great toe, with an ill‐defined erythematous margin and tenderness extending past the margin with intact overlying skin. His oral examination revealed multiple and painful vesicular lesions on the tongue and hard palate. Examination of other systems including the nervous system was unremarkable.

His blood counts revealed an absolute neutrophil count (ANC) of 0.0 and absolute lymphocyte count (ALC) of 0.8 × 10^9^/L. He was treated with empiric broad‐spectrum intravenous antibiotics (vancomycin and piperacillin/tazobactam), acyclovir, and filgrastim (a recombinant GM‐CSF) 480 micrograms for neutropenic fever. Evaluation for possible sources of infection with chest X‐ray, blood culture, and urine culture revealed no identifiable source of active infection. Evaluation for immunological markers of viruses was negative for Epstein‐Barr virus (EBV), cytomegalovirus (CMV), parvovirus B19, hepatitis B, and hepatitis C. His serum vitamin B12 was within normal limits. His ANC rose to 2.7 × 10^9^/L (Table 1) after administration of filgrastim and empiric antimicrobial treatment. To avoid the immediate effect of filgrastim on bone marrow, he had a bone marrow biopsy 1 month later, which showed mild nonspecific dyserythropoietic and mild megakaryocytic atypia, not suggestive of any primary bone marrow dysfunction. He also had flow cytometry and immunohistochemistry, which were nondiagnostic. He decided to continue the treatment of MS with ocrelizumab under close supervision. His next dose of ocrelizumab was scheduled 2 months after this hospitalization. However, he was found to have an ANC of 0.2 × 10^9^/L and ALC 0.5 × 10^9^/L. He received 1 dose of filgrastim, and his ANC rose to 2.1 × 10^9^/L.

**TABLE 1:** Serial blood workup before and after the commencement of therapy with ocrelizumab

	7/16/2019	1/29/2020 (Filgrastim given)	1/30/2020	1/31/2020	2/27/2020	4/3/2020 (Filgrastim given)	4/4/2020	4/5/2020	4/6/2020	1/19/2021
WBC	5.5	3.7	3.6	8	5.1	2.6	5.1	9.6	8.3	5.4
ANC	3.7	0	0	2.7	3.7	0.2	2.1	6.3	5.4	3.8
ALC	0.7	0.8	0.8	1	0.6	0.5	0.6	0.7	1	0.9
AMC	0.8	2.8	2.6	3.9	0.5	1.8	2.2	2.2	1.6	0.6
RBC	5.23	5.1	4.7	4.57	4.85	5.02	4.48	4.69	4.6	4.8
Hb	16.1	15.4	14.2	13.8	14.4	14.9	13.5	13.9	13.7	14.8
Hct	45.5	44.2	40.4	39.1	41.5	43.6	38.5	40	39.4	42.8

Note

All values are ×10^3^/µl (×10^9^/L).

Last dose of ocrelizumab‐1/8/2020.

Abbreviations: ALC, absolute leukocyte count; AMC, absolute monocyte count; ANC, absolute neutrophil count; Hb, hemoglobin; Hct, hematocrit; RBC, red blood cells; WBC, White blood cells.

## 3 DISCUSSION AND CONCLUSIONS

Late‐onset neutropenia is defined as ANC<1.5 × 10^9^/L with onset more than 4 weeks after the last drug administration, with a preceding normal ANC in absence of other identifiable causes. Ocrelizumab acts by targeting the CD20 receptor, which is a membrane‐glycosylated phosphoprotein expressed on pre‐B cells and mature B cells, leading to profound B‐cell depletion.^5^ The occurrence of neutropenia from PPMS clinical trials was 13% compared to 10% in the placebo group.^6^ The majority of the patients were found to have an ANC between 1.5 × 10^9^/L‐1.0 × 10^9^/L. Only 1% of the study population showed an ANC of <1.0 × 10^9^/L.^7^ While there is a paucity of data reporting LON with the use of ocrelizumab, the incidence with rituximab has been found to be 3%–27%.^8^ This variability in incidence may be due to a difference in the definition of LON that was used in previous studies.^7^

The mechanism of LON is poorly understood but various possible mechanisms have been proposed. Studies with bone marrow suggest white cell line maturation arrest with high B‐cell activating factor levels suggesting that bone marrow preferentially switches to B‐cell production possibly causing neutropenia.^8^ Other postulated mechanisms include infectious etiology, neutrophil Fc receptor FCγRI (CD64)‐mediated direct cell toxicity, and immune‐mediated neutropenia.^8^

The onset of LON after the last administered dose of ocrelizumab has been found to occur from 2.5 months to 10 months.^4^, ^5^, ^9^, ^10^ This is in accordance with our case where the onset is 3 months after the last dose. There have been only two reported cases presenting with fever and other infections in association with absolute neutropenia as in our case.^5^, ^10^ Others were diagnosed with decreased ANC on routine blood counts.^4^, ^9^ Search for active infection with blood, urine, and throat cultures and chest X‐rays should be performed in patients with a neutropenic fever for evaluation of neutropenia for the guidance of appropriate antimicrobial therapy.

There are other causes of acquired neutropenia such as viral infections (EBV, Parvovirus B19), immune‐mediated destruction, hematological malignancies (myelodysplastic syndromes), and nutritional deficiencies (B12 deficiency) that must be ruled out.^11^

Current literature suggests treatment with empirical antibiotic therapy and granulocyte colony‐stimulating factor (G‐CSF) provides improved outcomes as seen in our case. While there may be a difference in recommendations in the previously published literature regarding the need for G‐CSF in the treatment of LON as it is believed to accelerate disease course, it does in fact improve clinical outcomes.^12^ Our report supports existing literature that it does in fact assist in the resolution of the disorder as the ANC rose shortly after administration of filgrastim. It is important to note, however, that there are reports of possible flare‐ups of MS after G‐CSF administration, which may complicate its use in LON associated with ocrelizumab. Nonetheless, it may prove to be beneficial in treating patients experiencing LON due to ocrelizumab as the benefits that outweigh the risks as noted in our report.^13^

In conclusion, late‐onset neutropenia is a rare side effect of ocrelizumab treatment in MS patients. To the best of our knowledge, this is only the fifth reported case of LON associated with ocrelizumab. These neutropenic episodes may intermittently occur in patients on ocrelizumab, possibly triggered by a bacterial (or viral) infection. This is a rare unpredictable adverse event and outlines the importance of regular routine blood workup for detecting severe neutropenia early. The authors also highlight the importance of counseling patients to seek early medical care in the event the patient experiences fever or infections.

## CONFLICTS OF INTERESTS

The authors declare that they have no competing interests.

## AUTHOR CONTRIBUTION

RR2 (Rahul Rauniyar) and SA were involved in direct patient care and helped in reviewing the manuscript. RR1 (Robin Rauniyar), AA, and SY did the literature search and drafted the manuscript. All authors read and approved the final manuscript.

## CONSENT

Written informed consent was obtained from the patient to publish this report in accordance with the journal's patient consent policy.

## Data Availability

The datasets used and/or analyzed during the current study are available from the corresponding author on reasonable request.
